# Editorial: Current research and future development of neuropsychology

**DOI:** 10.3389/fnins.2025.1754906

**Published:** 2025-12-17

**Authors:** Aneta Szymaszek, Szczepan Iwanski, Aleksandra Bala

**Affiliations:** 1 Centre of Excellence for Neural Plasticity and Brain Disorders, BRAINCITY, Nencki Institute of Experimental Biology, Polish Academy of Sciences, Warsaw, Poland; 2 Department of Neurology, Institute of Psychiatry and Neurology, Warsaw, Poland; 3 Faculty of Psychology, University of Warsaw, Warsaw, Poland

**Keywords:** artificial intelligence, cognition, COVID-19, ecologically-valid assessment, neuromodulation, neuropsychological assessment, virtual reality

Neuropsychology is undergoing a rapid transformation, integrating advances from cognitive neuroscience, neuroimaging, computational modeling, and clinical practice. The aim of this Research Topic, *Current research and future development of neuropsychology*, was to bring together innovative approaches and empirical findings that illustrate how the field is evolving toward greater precision, ecological validity, and translational relevance. The articles collected here reflect this diversity and ambition. They address fundamental and applied questions, ranging from the identification of early cognitive decline and novel biomarkers, through the development of immersive and AI-based assessment tools, to the exploration of targeted neuromodulation and post-COVID-19 neuropsychological consequences. Together, they highlight emerging directions and methodological advances that shape contemporary neuropsychology.

A key theme across several contributions is the relationship between brain structure, connectivity, and cognition. One of the studies explored patients with left mesial temporal lobe epilepsy (Olejnik et al.). Structural abnormalities in specific brain regions were linked to memory impairment, reinforcing the connection between focal pathology and neuropsychological outcomes. Complementary evidence from resting-state EEG research shows altered alpha-band connectivity in individuals with occupational burnout, identifying potential electrophysiological markers that could help differentiate burnout from related affective disorders (Afek et al.). Another contribution combines transcranial direct current stimulation (tDCS) with language therapy in patients with chronic non-fluent aphasia (Alemanno et al.). In this work, anodal stimulation of the right hemisphere enhanced interhemispheric connectivity and was associated with language improvements, pointing to the adaptive role of the right hemisphere in language recovery.

A significant methodological trend represented in this Research Topic is the development of immersive and ecologically valid assessment tools. A systematic review of virtual reality (VR)-based memory assessments demonstrates strong convergence with traditional neuropsychological tests, while offering improved ecological validity by simulating real-world contexts and engaging executive functions (Mancuso et al.). Another article illustrates how VR can serve both diagnostic and therapeutic purposes, using autism spectrum disorder as an example (Sokołowska et al.). Controlled virtual environments can support individualized assessments and interventions, offering adaptive social scenarios and detailed behavioral data.

Parallel to these developments, advances in artificial intelligence (AI) are reshaping neuropsychological assessment. One contribution presents an interpretable deep learning model capable of detecting driver drowsiness from single-channel EEG recordings (Feng et al.). The model combines high classification performance with explainability, a key requirement for clinical adoption. Another perspective outlines a roadmap for integrating AI into neuropsychology responsibly, emphasizing the importance of transparency, bias mitigation, and rigorous validation (Lundervold). These approaches demonstrate how machine learning can enhance neuropsychological practice while maintaining interpretability and ethical standards.

Another important research diagnostic area is the early detection of cognitive decline and the refinement of diagnostic tools. One study presents a multidimensional screening battery designed for individuals with subjective cognitive decline. The results emphasize the importance of integrating affective and cognitive components, as depressive symptoms were found to contribute significantly to subjective complaints. The new tool demonstrates promising psychometric properties and may support earlier and more accurate identification of individuals at risk of cognitive deterioration (Maffoni et al.).

The neuropsychological consequences of COVID-19 are addressed in two contributions. One article examines the role of the olfactory system in cognitive and neuropsychiatric symptoms following infection, proposing mechanistic links between sensory dysfunction, limbic connectivity, and cognitive-affective outcomes (Azcue et al.). Another study identifies psychosocial factors associated with prolonged post-traumatic stress symptoms after mild COVID-19, suggesting potential targets for early intervention and prevention (Elkayam et al.).

The interplay between spontaneous cognition, sleep, and affect is another topic addressed in this Research Topic (Fell). One perspective proposes a conceptual framework linking mind wandering, poor sleep, and negative affect in a self-reinforcing cycle. This integrative view underscores the need to consider dynamic interactions among cognitive control, arousal, and mood regulation mechanisms in clinical practice and suggests avenues for future intervention strategies.

The final, yet highly significant, component of the Research Topic is the cognitive intervention supported by neuromodulation methods. The potential of tDCS in healthy and clinical populations is examined in two studies. In one, the effects of multisession tDCS on memory-related cognitive functions are explored in healthy young adults, contributing valuable data on the boundary conditions of stimulation efficacy (Kukuła et al.). In another, the aforementioned study in patients with aphasia demonstrates that targeted stimulation, informed by network-level insights, may yield functional improvements, supporting the move toward personalized neuromodulation protocols (Alemanno et al.). This study also indicates the importance in combining neuromodulation methods with behavioral therapy to achieve the highest possible improvement in the clinical population.

Summarizing the articles in this Research Topic collectively showcase the dynamic and interdisciplinary nature of contemporary neuropsychology. Several cross-cutting themes emerge from this body of work. First, the importance of network-level mechanisms is increasingly evident. Studies examining structural and functional connectivity demonstrate that cognitive and clinical phenomena are better understood within the context of distributed neural networks rather than isolated regions. This systems-level perspective informs both assessment and intervention strategies, including the design of neuromodulation protocols tailored to individual network profiles.

Second, there is a growing emphasis on ecological validity and multidimensional assessment. Tools such as VR-based tasks and EEG-based biomarkers capture aspects of cognition and behavior that traditional assessments may overlook, providing richer insights into everyday functioning. This shift is crucial for developing diagnostic and therapeutic strategies that translate effectively into real-world contexts.

Third, the integration of artificial intelligence offers new opportunities for scalable, data-driven neuropsychology. However, the contributions in this Research Topic emphasize that interpretability, validation, and ethical considerations are essential to ensure that AI tools are clinically useful and trustworthy.

Finally, the work presented here highlights the necessity of addressing affective, sleep-related, and sensory dimensions alongside cognitive processes. These factors interact dynamically and shape neuropsychological outcomes, particularly in emerging contexts such as post-COVID-19 conditions.

Looking ahead, the field is poised to advance through closer integration of neuroimaging, electrophysiology, immersive technologies, and computational modeling. Future research should aim to validate novel tools across diverse populations and contexts, link biomarkers to specific cognitive and behavioral outcomes, and develop personalized intervention strategies that leverage network-level insights. By doing so, neuropsychology can continue to evolve into a discipline that is not only scientifically rigorous but also clinically impactful and socially relevant.

